# A Nursing Care Intervention Model for Elderly People to Ascertain General Profiles of Functionality and Self Care Needs

**DOI:** 10.1038/s41598-020-58596-1

**Published:** 2020-02-04

**Authors:** Margarida Goes, Manuel José Lopes, Henrique Oliveira, César Fonseca, João Marôco

**Affiliations:** 1 Escola Superior de Saúde, Instituto Politécnico de Beja, Beja, Portugal; 2 Escola Superior de Enfermagem de São João de Deus, Universidade de Évora, Évora, Portugal; 3 Instituto de Telecomunicações, Lisbon, Portugal; 4 ISPA - Instituto Universitário de Ciências Psicológicas Sociais e da Vida, Lisbon, Portugal

**Keywords:** Geriatrics, Rehabilitation

## Abstract

A core set of International Classification of Functioning, Disability and Health codes was used to ascertain general profiles of functionality as a function of biological and sociodemographic characteristics and to develop structured nursing interventions in accordance with self care deficits identified by studying self care behavior for elderly people living in both extensively and sparsely populated rural areas. Data were collected by health professionals in the participants’ houses. An exploratory factor analysis enabled reduced data dimensions, and factorial validity was assessed by a confirmatory factor analysis. An ordinal regression model was built to identify general profiles of functionality as a function of age. A bar graph was used as a measurement tool for nursing care needs as a function of self care behavior and functional profile level. No functional problems were expected among people under the age of 74 years, while mild functionality problems were expected among people older than 74 years. Regarding nursing care needs, the results of the constructed model suggested that the functional concept “*Support and Relationships*” is associated with higher levels of functional problems and thus a greater need for self care interventions and that people aged 85 years and older always show therapeutic self care deficits.

## Introduction

Portugal has one of the highest aging indexes for the resident population among European Union countries (153.2%)^1^, and national estimates indicate that this index will more than double by 2080^2^. The Baixo Alentejo region (BAR), which is the setting for this study, is predominantly rural, has the lowest population density in the country and shows a worrisome aging index (189.2%)^3^. The region is characterized by extensive distances between villages (with some villages located more than 120 km apart by road) and a limited and inefficient public transportation network. Since the region is large and 25% of the residents are over 65 years old, some of whom presenting disabilities and self-care deficits, a quick and effective access to health services within a reasonable time-frame may become problematic, especially among elderly who cannot travel easily, with these persons becoming more vulnerable when compared to other age-groups of the population^4^.

Some of the disabilities that emerge with aging^5^ are not always directly related to diseases, and senility and senescence must be distinguished as two distinct processes, with the former referring to pathological aging and the latter referring to the normal aging process^6^. Thus, asserting that aging is inevitably associated with an increased probability of developing various chronic diseases is not completely accurate. Disabilities often result from situations encountered throughout life and can be modifiable. However, human aging is a slow and inevitable process, leading to various morphological, functional, biochemical and psychological changes that may contribute to increasing vulnerability to and the incidence of pathological processes in the body. In most cases, elderly patients experiencing worsening health conditions (e.g., cardiac, respiratory or cerebrovascular problems, urinary infections, diabetes and fall-related injuries) usually use the emergency department of the Hospital of BAR, according to several international studies^4,7–9^.

The Ministry of Health of Portugal, the European Observatory on Health Systems and Policies and the Regional Office for Europe of the World Health Organization (WHO) initiated an assessment of the Portuguese health system’s performance in addressing key challenges and opportunities in the post-financial crisis recovery period. The report states that the estimated overall burden of chronic diseases in the Portuguese population (regardless of age/gender) is as follows: (i) 18% of Portuguese users of the National Health System (Sistema Nacional de Saúde - SNS) have one chronic disease, (ii) 11% of users have two chronic diseases, (iii) 8% of users have three chronic diseases, and (iv) 22% of users have four or more chronic diseases; therefore, at least 59% of the Portuguese population has one or more chronic diseases (estimate). Additionally, the same report reveals that multimorbidity increases with age and peaks (80% of the population has more than one chronic disease) between 80 and 90 years of age. Furthermore, between 10 and 90 years of age, multimorbidity is always more common among women than among men, although this finding is reversed for people older than 90 years^1^.

Due to the complexity and heterogeneity of individual aging, the level of functionality may vary distinctly from person to person. Studying individual aging by integrating a continuous and accessible care model that allows elderly individuals as well as family caregivers to monitor and manage their health at home—always under the supervision of health professionals, which may enable the management of various chronic conditions (multimorbidity) and provide a “safety net” before a health crisis requiring emergency department care occurs^2^—was the main motivation behind the present study^3^. To achieve this goal and to promote improved health-related quality of life among elderly individuals by requalifying their potential and allowing them to live more autonomously, the following tasks are essential: (i) evaluation of individuals’ functional capacity to identify their disabilities and (ii) identification of appropriate self care behavior that allows determining, planning and assessing preventive nursing care needs^4^. According to Lesende *et al*.^5^, health care for patients with multimorbidity and applicable intervention strategies yield better outcomes when structured based on a previous evaluation of the patient’s functional state.

By providing multidisciplinary interoperability, the WHO has developed several tools to devise a standardized international health information system. One example is the International Classification of Functioning, Disability and Health (ICF)^6^. This classification encompasses “bio-psycho-socio-environmental” factors because it classifies (i) the functioning, disability and health of a person as an interrelationship among health states, (ii) bodily functions and structures (i.e., the presence or absence of disabilities), (iii) activity (i.e., the execution of a task or an individual’s actions), (iv) participation (i.e., involvement in real-life situations) and (v) contextual (i.e., environmental and personal) factors that can act as “barriers” or “facilitators”^7–9^.

**Figure 1:**
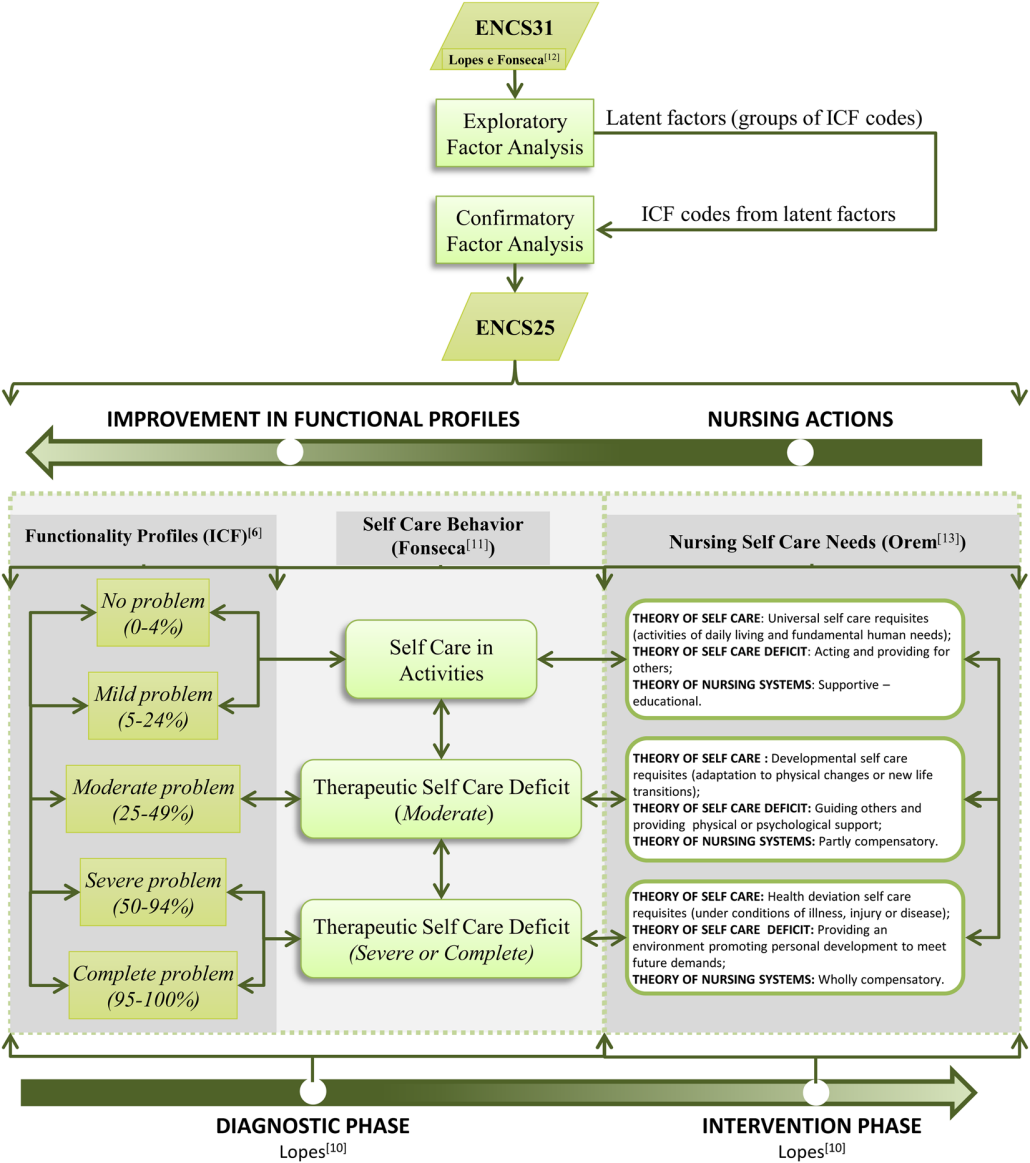
Synthesis of the conceptual framework of the ENCS25 nursing model and the correspondence between functional profiles and nursing self care deficits according to the self care behavior of a person aged 65 or older within his or her residence, namely, his or her home or the home of family or friends.

In Portugal, Lopes^10^ and then Fonseca^11^ developed a tool to evaluate the individual functionality profiles of people aged 65 and older called the Elderly Nursing Core Set (ENCS), which resulted in a set of 31 items (ICF codes) hereafter referred to as the Portuguese “ENCS31” (see the 1^st^ top block symbol in dark green in Fig. 1), thus providing a more feasible method for evaluations of functionality^12^.

The conceptual framework of the self care nursing model for people aged 65 and older proposed in this article is based on (i) the medium-range theory proposed by Lopes^10^, (ii) the self care deficit theory proposed by Orem^13^ and (iii) the functionality/disability continuum proposed by Fonseca^11^. Lopes^10^ described two crucial phases in the context of a health care system, namely (see Fig. 1), the diagnostic phase and the intervention phase. However, Fonseca^11^ established the following theoretical associations between self care deficits (using an ordinal scale ranging from 1 to 3) and functional profiles of the ICF (using a Likert (LK) scale ranging from 1 to 5) (see Fig. 1): (i) “self care in activities” is associated with “no problem (0–4%)” and “mild problem (5–24%)” functional profiles, (ii) “therapeutic self care deficit (moderate)” is associated with the “moderate problem (25–49%)” functional profile, and (iii) “therapeutic self care deficit (severe or complete)” is associated with both “severe problem (50–95%)” and “complete problem (96–100%)” functional profiles. Therefore, in the first phase—the diagnostic phase—a nurse evaluates a person’s condition and his/her functional profile. After a functional evaluation is performed, the nurse considers different self care deficits, namely, (i) self care in activities, (ii) a moderate therapeutic self care deficit and (iii) a severe or complete therapeutic self care deficit. After the diagnostic phase of an elderly person, the second phase begins—the intervention phase—where a nursing intervention is selected according to the diagnosed self care deficit. Examples of nursing interventions based on Orem’s self care theory^13^ can be found in the three rounded boxes containing information regarding self care deficits on the right in Fig. 1.

Since the previous research tool for functionality was designed only for institutionalized elderly persons^12^, the authors of the present research applied the Portuguese ENCS31 to people aged 65 or older residing in the community to complete the following tasks: (i) extract (through an exploratory factor analysis, EFA) a set of latent factors that explain the relational structure of a core set items and apply them to community residents, (ii) validate the model derived from the EFA through a confirmatory factor analysis (CFA), (iii) calculate descriptive statistics for biological and sociodemographic variables and the proportions of various general profiles of functionality (GPFs), (iv) standardize the GPFs based on age and sex and (v) define nursing care needs for elderly persons based on the mean levels of the latent factors according to self care behaviors as a function of age group. Therefore, the disability level can be assessed through a functionality assessment, enabling subsequent determination of health care needs in all dimensions of disability, including body functions and structures, activities and participation as well as associated impairments, limitations, restrictions and environmental factors. The current model was built from the work developed for the 4IE project (http://www.4ie.uevora.pt/objectivos/), the data and results of which integrate the platform developed as part of the project “Enriched Elderly Virtual Profiles By Means Of A Multidimensional Integrated Assessment Platform”^14^.

Thus, the objective of this study is to propose a conceptual framework to assess the nursing care needs of specific population subgroups according to self care deficits. The main tasks of the model include (i) determining the probability that an elderly person has a specific GPF as a function of age regardless of gender and (ii) identifying the real nursing care needs of the sample in average terms and for each age group with respect to each concept of functionality or GPF.

In summary, the present research study aims to answer the following research questions:

- “Are GPFs identical between men and women?”
- “Do GPFs vary with age?”
- “What are the predicted average nursing care needs of elderly individuals residing in the BAR by age group?”

## Methods

### Subjects

The ULSBA’s Health Ethics Committee (in the Portuguese language: Comissão de Ética para a Saúde da Unidade Local de Saúde do Baixo Alentejo–HECULSBA) served as the institutional review board that approved the study protocol, including the study’s design and execution, the method and format used for the interviews and the informed consent form that was presented to each person. Full information and the composition of the Institutional Review Board of ULSBA, including the names of professionals, can be found at the HECULSBA webpage^15^. Moreover, all methods used in our research were performed in full accordance with all the statements included in the operating regulations of HECULSBA, which are available at the corresponding webpage^16^; these regulations were developed under the Helsinki Declaration to protect the dignity, privacy and freedom of study participants^17^.

This study examined a population of individuals aged 65 years or older who were registered in the Portuguese Health System of the BAR, namely, the Local Health Unit of Baixo Alentejo (ULSBA for its acronym in Portuguese)^18^. The sample size was calculated using the formulae proposed by Scheaffer *et al*.^19^, and the participants were stratified by gender (male and female) and age group (65 to 74 years, 75 to 84 years and 85 years or older) using Neyman optimal allocation based on all elderly individuals listed in the ULSBA database (32,893). The calculated sample size was 470 elderly individuals randomly selected from the ULSBA database. The inclusion criteria were as follows: (i) 65 years of age or older, (ii) a desire to participate in the study, (iii) residence in the BAR in one’s own home or in the home of family members or friends and (iv) and the ability to make independent decisions even if sick or hospitalized. The final (random) sample stratified by gender (male and female) and age group (65 to 74 years, 75 to 84 years and 85 years or older) included 351 people who fulfilled all the inclusions criteria, signed the informed consent document and answered all the ENCS31 questions.

Data were collected between January 2016 and April 2017 at the homes of the participants by health teams from ULSBA using the ENCS31. Prior to each interview, a health professional presented the informed consent document (which was specifically developed for this study and previously approved by the ethics committee of ULSBA) to the interviewee and his or her family. The informed consent document was read completely by the interviewee in the presence of the health professional or by the health professional if the interviewee was unable to read it. Complete information about the study objectives was provided to the individuals and their families, including a statement that their confidentiality and anonymity would be guaranteed. The interview was initiated only after the elderly individual agreed to participate in the study and freely signed the informed consent form and lasted at least 30 to 45 minutes, depending on the interviewee’s age and his or her level of literacy. However, every individual was allowed to withdraw from the interview at any time at his or her own discretion.

### Statistical procedures

Figure 1 details the synthesis of the conceptual framework of the proposed nursing model. To identify latent factors that explained the relational structure of the 31 items included in the ENCS31, an EFA (see the 2^nd^ top block symbol in light green color in Fig. 1) based on a correlation matrix, was conducted. Factors were extracted via principal component analysis using varimax rotation. Subsequently, factors with an eigenvalue greater than 1 were retained in the model. The validity of the EFA was assessed using the Kaiser-Mayer-Olkin (KMO) criterion, and the quality of the fitted model was assessed using the goodness-of-fit index (GFI), adjusted goodness-of-fit index (AGFI) and modified root mean square residual (RMSR*) as suggested by Marôco^20^.

A descriptive analysis was performed to describe the biological and sociodemographic variables of the sample and the GPF scores of the 25 items identified by the EFA using absolute and relative frequencies.

The factorial validity of this new subset of items—the “ENCS25”—was assessed through CFA (see the 3^rd^ top block symbol in light green color in Fig. 1) using SPSS AMOS version 23.0.0 (IBM, Armonk, NY) as described by Marôco^21^. The CFA included the following steps: (i) construct reliability was evaluated using Cronbach’s alpha (α) and composite reliability (CR) as an alternative measure, (ii) construct validity was evaluated by analyzing the factor weights of the model (factorial validity), and (iii) the average variance extracted (AVE) was calculated for each latent factor (convergent validity, CV), and squared correlation coefficients were compared between the latent factors (discriminant validity). The following indexes were also used to evaluate the quality of the CFA model as described by Marôco^21^: (i) chi-squared statistics divided by the degrees of freedom (${{\rm{\chi }}}^{2}/{\rm{df}}$), (ii) the comparative fit index (CFI), (iii) the parsimony comparative fit index (PCFI); (iv) the GFI, (v) the parsimony goodness-of-fit index (PGFI), (vi) the root mean square error of approximation (RMSEA), (vii) the “p value” for testing the null hypothesis that the RMSEA is less than 0.05 in the population (PCLOSE) and (viii) the mean expected cross-validation index (MECVI), which was used to compare adjusted models, and the model with the lowest MECVI value considered the most stable model in the population. Because the maximum likelihood method was used to estimate the CFA model parameters, the assumption of normality was tested by analyzing skewness and kurtosis values. The overall goodness-of-fit of the model was assessed based on the indexes suggested by Marôco^21^. The items with the highest weights in the estimation of the scores of the latent factors were identified through an analysis of factor score weights (*fsw*). Finally, the validation process confirmed the 25 items extracted from the ENCS31^12^, which were collectively called the ENCS25 (see the 4^th^ top block symbol in dark green color in Fig. 1).

An ordinal regression model was then used to evaluate whether the ages and genders of the respondents had a significant effect on the GPFs (which included the 25 items extracted by the EFA) and to answer the research questions “Are GPFs identical between men and women?” and “What is the effect of age?”

Subsequently, the means of the functional profiles of each latent factor and the GPFs, stratified by age group, were calculated. Based on these results and on the association between the functional profiles (measured using an ordinal scale ranging from 1 to 5) and Orem’s “nursing self care deficits”^13^ in accordance with self care behavior^11^ (measured using an ordinal scale ranging from 1 to 3), a bar plot was constructed to visualize the answer to the research question “What are the predicted average nursing care needs of elderly individuals residing in the BAR by age group and latent factor?”

## Results

### Exploratory factor analysis

Table 1 lists the final values of the EFA results (only factor weights above 0.49 are shown). Because of the high skewness and kurtosis values of some items, the analysis was based on Spearman’s correlation matrix rather than the original variables. Using the “eigenvalue greater than 1” rule, the relational structure of the items was explained by five latent factors (“Self care in activities of daily living (a)” – SC-ADL(a), “Self care in activities of daily living (b)” – SC-ADL(b), “Mental functions” – MF, “Communication” – COM and “Support and relationships” – SR), with only two factor weights at a threshold of 0.5 (“Toileting” and “Higher-level cognitive functions”). Six items (“Sensation of pain”, “Blood pressure functions”, “Respiration functions”, “Respiratory muscle functions”, “Defecation functions” and “The structure of areas of skin”) were iteratively removed, thereby reducing the number of latent factors from eight to five. The final model (referred to as the ENCS25) was “excellent” based on the recommendation derived from the EFA (KMO = 0.909). The total variance explained by the model was 64.3%, and the first latent factor explained more than half of the total variance. In general, almost all communalities were high (only 20% of the values were less than 0.5, and none of the values were below than 0.3, with this last value being considered as an appropriate threshold in exploratory studies^20^. The average of the factor weights belonging to SR latent factor was low (an average of factor weights within each latent factor of 0.7 or higher is preferable, but a value of only 0.583 was obtained for the SR factor), which may compromise the correlational structure between the items of this latent factor. This finding seems to be reflected by the values obtained for the goodness-of-fit indexes (GFI = 0.630 and AGFI = 0.350) to some extent, although a “very good” RMSR* of 0.05 was achieved. The reliability of each latent factor was evaluated based on Cronbach’s alpha, and the values ranged from “very good” to “reasonable” except for the latent factor SR (<0.60 but very close to the threshold for “weak”).

**Table 1:** The EFA and CFA results: (i) a list of the five retained latent factors and their respective factor weights, eigenvalues and percentages of variance explained.

Items (ICF codes)	Latent factors*					Com.	*fsw*	Mean (LK scale)	PF***
	SC-ADL (a)	SC-ADL (b)	MF	COM	SR				
Walking	0.807	—	—	—	—	0.716	0.086	1.55	Mild
Changing the basic body position	0.804	—	—	—	—	0.747	0.019	1.56	Mild
Moving around using equipment	0.763	—	—	—	—	0.666	0.092	1.47	Mild
Maintaining a body position	0.761	—	—	—	—	0.646	0.075	1.43	Mild
Carrying out a daily routine	0.753	—	—	—	—	0.703	0.119	1.76	Mild
Washing oneself	0.651	—	—	—	—	0.720	0.068	1.45	Mild
Caring for body parts	0.649	—	—	—	—	0.714	0.168	1.44	Mild
Dressing	0.568	0.555	—	—	—	0.711	0.181	1.31	Mild
Eating	—	0.808	—	—	—	0.791	0.398	1.11	No
Drinking	—	0.781	—	—	—	0.772	0.461	1.08	No
Toileting	—	0.492	—	—	—	0.508	0.035	1.29	Mild
Orientation	—	—	0.816	—	—	0.792	0.160	1.23	Mild
Consciousness	—	—	0.809	—	—	0.795	0.125	1.23	Mild
Attention	—	—	0.795	—	—	0.761	0.315	1.26	Mild
Emotional functions	—	—	0.587	—	—	0.489	0.077	1.41	Mild
Memory	—	—	0.570	—	—	0.523	0.031	1.79	Mild
Higher-level cognitive functions	—	—	0.495	—	—	0.595	0.018	1.71	Mild
Conversation	—	—	—	0.799	—	0.821	0.511	1.18	Mild
Speaking	—	—	—	0.741	—	0.751	0.256	1.13	No
Communicating with and receiving spoken messages	—	—	—	0.662	—	0.713	0.143	1.27	Mild
Immediate family	—	—	—	—	0.718	0.548	0.089	1.58	Mild
Friends	—	—	—	—	0.644	0.469	0.054	2.21	Moderate
Health professionals	—	—	—	—	0.533	0.336	0.074	1.57	Mild
Personal care providers and personal assistants	—	—	—	—	0.514	0.390	0.179	1.20	Mild
Family relationships	—	—	—	—	0.504	0.403	0.195	1.19	Mild
Mean	***0.720***	***0.694***	***0.679***	***0.734***	***0.583***	***0.643***	—	***1.416***	
*Eigenvalue*	9.749	1.606	2.156	1.438	1.130	—	—	—	
*Variance explained*	39.0%	6.4%	8.6%	5.8%	4.5%	—	—	—	
α**	0.942 (Very Good)	0.731 (Reasonable)	0.824 (Good)	0.898 (Good)	0.545 (Almost Unallowable)	—	—	—	

Groups of vertical cells on a gray background represent one retained latent factor; (ii) Communalities (Com.) and *fsw* values extracted from the CFA, with the average results presented in terms of a Likert (LK) scale, and the corresponding profile of functionality (PF).

*Extraction Method: Principal component analysis.

Rotation Method: Varimax with Kaiser normalization.

Rotation converged in seven iterations.

**Qualitative classification adopted from Marôco^20^.

***Labeled according to the mean LK scale score for each code, after correspondence with the ICF scale of profiles was confirmed.

### Confirmatory factor analysis

The ENCS25 was subjected to a CFA. The initial model without correlations between the measurement errors of any items showed a “fair” overall quality of fitting (see Table 2). However, after correlating the measurement errors of “Walking” with “Moving around using equipment (wheelchair, skates, etc.)”, “Changing the basic body position” with “Maintaining a body position” and “Carrying out a daily routine”, “Washing oneself (bathing, drying, washing hands, etc.)” with “Caring for body parts (brushing teeth, shaving, grooming, etc.)”, “Orientation (time, place, person)” with “Consciousness” and finally “Memory” with “Higher-level cognitive functions”, as suggested by the modification indices (MI > 11), the overall model goodness-of-fit was classified as “fair” to “reasonable”. Regarding the correlations between the above measurement errors of the items, the authors assume that all items present some similarity in their formulation and that their contents are related to a certain extent, such as “Washing oneself” (bathing, drying, washing hands, etc.) and “Caring for body parts” (brushing teeth, shaving, grooming, etc.) (see Table 2). A CFA simulation using only four latent factors by merging SC-ADL(a) and SC-ADL(b) into one latent factor was done. Results, without any correlation between items errors suggested by MI, show that this new model revealed worst global adjustment indexes (MECVI = 4.758) when compared to the initial model with five latent factors (see MECVI = 4.020 in Table 2), suggesting that the CFA model with five latent factors was considered as “more appropriate” than the one presenting the merge of SC-ADL(a) and SC-ADL(b) into SC-ADL.

**Table 2:** Results of the adjustment indexes for the initial and adjusted CFA models.

Indexes	Initial Model	Adjusted Model	Qualitative classification of the adjusted model*
${{\rm{\chi }}}^{2}/{\rm{df}}$	4.820	3.421	Reasonable
CFI	0.837	0.899	Almost Good
GFI	0.757	0.827	Reasonable
TLI	0.816	0.883	Reasonable
PCFI	0.739	0.776	Good
PGFI	0.617	0.659	Reasonable
Standardize RMR	0.0734	0.0678	Acceptable
RMSEA	0.104	0.083	Acceptable
RMSEA - IC_95%(Low)_	0.099	0.077	—
RMSEA - IC_95%(High)_	0.110	0.089	—
PCLOSE	<0.001	<0.001	—
MECVI	4.020	2.939	Better

*Based on the qualitative classification of Table 4.1 in Marôco^21^.

Of the 25 standardized factor weights, only two (12%) presented values lower than 0.5, suggesting that the factorial validity of the construct is considered “favorable”. The construct reliability was evaluated using CR measure (an alternative to Cronbach’s alpha as suggested by Marôco^21^ based on the results from the CFA). All CR values were greater than 0.8 (which is considered to reflect appropriate reliability of a construct since the value is higher than 0.7 as suggested by Marôco^21^) except for the “SR” latent factor (CR = 0.564), although values less than 0.7 may be accepted in exploratory research^22^ as in the present paper. Regarding convergent validity, the AVE values were always greater than 0.5, representing a “favorable” convergent validity as suggested by Marôco^21^, although a “weak” (AVE = 0.210) value was found again for the “SR” latent factor (an expected result since this latent factor includes three standardized regression weights lower than 0.5: 0.36, 0.37, 0.46, respectively).

The estimated *fsw* values of the ENCS25 items resulting from the CFA, the average answers for each item on a Likert (LK) scale and the corresponding functional profile can be found also in Table 1 (the three columns on the right). The analysis showed that some items have high *fsw* values while others are associated to lower ones; thus, changes in items presenting high *fsw* values will have a greater effect on the scores of the respective latent factors compared with items with lower *fsw* values, if a weighted average formulation (adopting *fsw* as weights) is used to compute the scores of each latent factor. Analyzing cases in which an item has a high mean value associated with a low *fsw* (e.g., “Friends”, with M = 2.21 and *fsw* = 0.054) is essential because it reveals that an important “moderate problem” may have a low impact (e.g., weight) when calculating the score of the “SR” latent factor since the respective *fsw* value (the weight in a weighted average formulation) of that ICF code (“Friends”) is low. On the other hand, two of the items in the latent factor “SC-HFN” present some of the highest *fsw* values (e.g., “Eating” and “Drinking”), indicating that these items have a stronger impact on the score calculation of “SC-HFN” latent factor. The highest value of *fsw* = 0.511 corresponded to the item “Conversation” of the “COM” latent factor. Using the quantitative correlation between the ICF five-profile scale (from 0% to 100%) and the Likert scale (1 to 5), a functional profile was obtained for each factor (see the rightmost column in Table 1).

### Biological and sociodemographic variables and GPFs

The sample data exhibited a higher proportion of women than men (Table 3). Most respondents were married, although widowers accounted for a considerable proportion of the sample (32.5%: 76.3% of women and 23.7% of men). Regarding education level, the eight categories listed were reduced to four because of the small absolute frequency observed for higher education levels. Approximately half of the respondents (46.4% = 29.6% + 16.8%) did not have a formal education, and 29.6% of the sample (57.8% of women and 42.2% of men) was illiterate. A quick review of the GPFs showed that the largest proportion of the “no problem (0–4%)” profile corresponded to individuals in the younger group and those with a higher education level. The most common profile observed was “mild problem (5–24%)” for almost all other variables (the numbers in bold font in Table 3 highlighted the highest proportions in each variable).

**Table 3:** This table lists the biological and sociodemographic characteristics of the 351 respondents residing in the BAR as well as the proportions of general profiles of functionality taken from the ENCS25.

Variables	*n*	%	General Profile of Functionality				
			NO 0–4%	Mild 5–24%	Moderate 25–49%	Severe 50–95%	Complete 96–100%
*Gender:*	—	—	—	—	—	—	—
Male	163	46.4	34.6%	**56.4%**	6.4%	2.7%	0.0%
Female	188	53.6	37.4%	**50.9%**	11.0%	0.6%	0.0%
*Age group:*	—	—	—	—	—	—	—
65–74	132	37.6	**58.3%**	37.9%	2.3%	1.5%	0.0%
75–84	135	38.5	27.4%	**62.2%**	8.9%	1.5%	0.0%
85 and older	84	23.9	14.3%	**65.5%**	17.9%	2.4%	0.0%
*Marital status:*	—	—	—	—	—	—	—
Single	27	7.7	25.9%	**59.3%**	14.8%	0.0%	0.0%
Married	206	58.7	42.2%	**49.5%**	6.3%	1.9%	0.0%
Divorced	4	1.1	0.0%	**100.0%**	0.0%	0.0%	0.0%
Widowed	114	32.5	28.1%	**58.8%**	11.4%	1.8%	0.0%
*Educational level:*	—	—	—	—	—	—	—
Does not know how to read or write	104	29.6	12.5%	**65.4%**	18.3%	3.8%	0.0%
Knows how to read and write	59	16.8	28.8%	**62.7%**	6.8%	1.7%	0.0%
1^st^-4^th^ grade	165	47.0	46.1%	**49.7%**	3.6%	0.6%	0.0%
More education	23	6.6	**87.0%**	8.7%	4.3%	0.0%	0.0%

### Standardizing the GPFs based on age and sex

**Figure 2:**
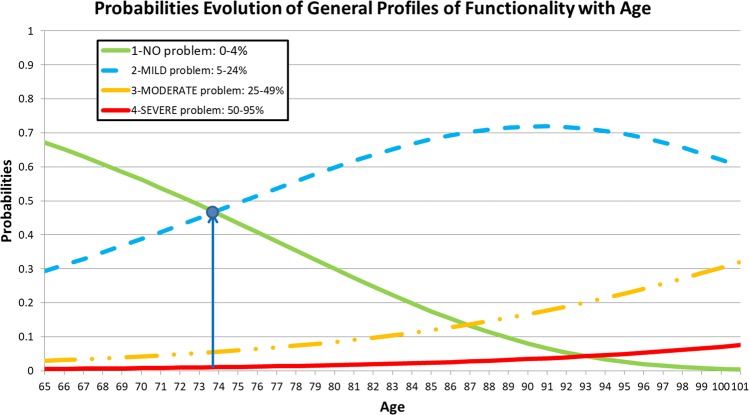
Probability evolution of the general profiles of functionality as a function of the ages of the respondents. The plot shows that for a person whose age is higher than 74 years old, he/she has a greater probability to present a “MILD problem” functional profile than a “NO problem”, whose result is statistically significant.

To evaluate whether the ages and genders of the respondents had a significant effect on the GPFs, an ordinal regression model was established. The results showed that gender was not significant in the model: (i) *b*_*Gender*_ = 0.045, with *p* = 0.750 and a 95% confidence interval (CI) equal to CI_95%_ = [−0.234, 0.325], and (ii) *b*_*Age*_ = 0.074, with *p* < 0.001 and CI_95%_ = [0.055, 0.093]; thus, no statistical evidence supports the hypothesis that GPFs differ between men and women. The model was again reproduced only for age. The final model was considered highly significant ($-2LL=193.832$, χ^2^(1) = 64,347, *p* < 0.001), although the effect size was slightly small (${R}_{CS}^{2}=0.168$; ${R}_{N}^{2}=0.195$; ${R}_{MF}^{2}=0.093$). In the ordinal regression model, the link function “Log-negative log” $P(Y\le K)=exp(-\exp (-({b}_{K}-{b}_{Age}\times Age)))$ was adopted because this function is recommended when the classes of the lowest-order dependent variable (functional profiles indicating a less severe problem) show a higher frequency compared to the classes of severe and complete problems (presenting low frequencies)^21^. *b*_*K*_ is the coefficient associated with each of the three thresholds obtained because no respondent showed a “complete problem” GPF (*b*_*K* = 1_ = 5.718, *p* < 0.001, *b*_*K* = 2_ = 8.138, *p* < 0.001 and *b*_*K* = 3_ = 9.994, *p* < 0.001). As age increased, the probability of observing more severe functional problems increased, because the parameter estimate for age was positive (*b*_*Age*_ = 0.074, *p* < 0.001; see the curves in Fig. 2). For the GFI of the model, Pearson’s chi-squared and deviance tests revealed that the null hypothesis regarding the model fit was not rejected (${{\rm{\chi }}}_{{\rm{Pearson}}}^{2}(98)=90.232$, p = 0.699 and ${{\rm{\chi }}}_{{\rm{Deviance}}}^{2}(98)=79.202$, *p* = 0.918). The assumption of slope homogeneity was validated ($-2{\rm{LL}}=191.031$, χ^2^(2) = 2.801, *p* = 0.246).

### Nursing care needs for elderly individuals

**Figure 3:**
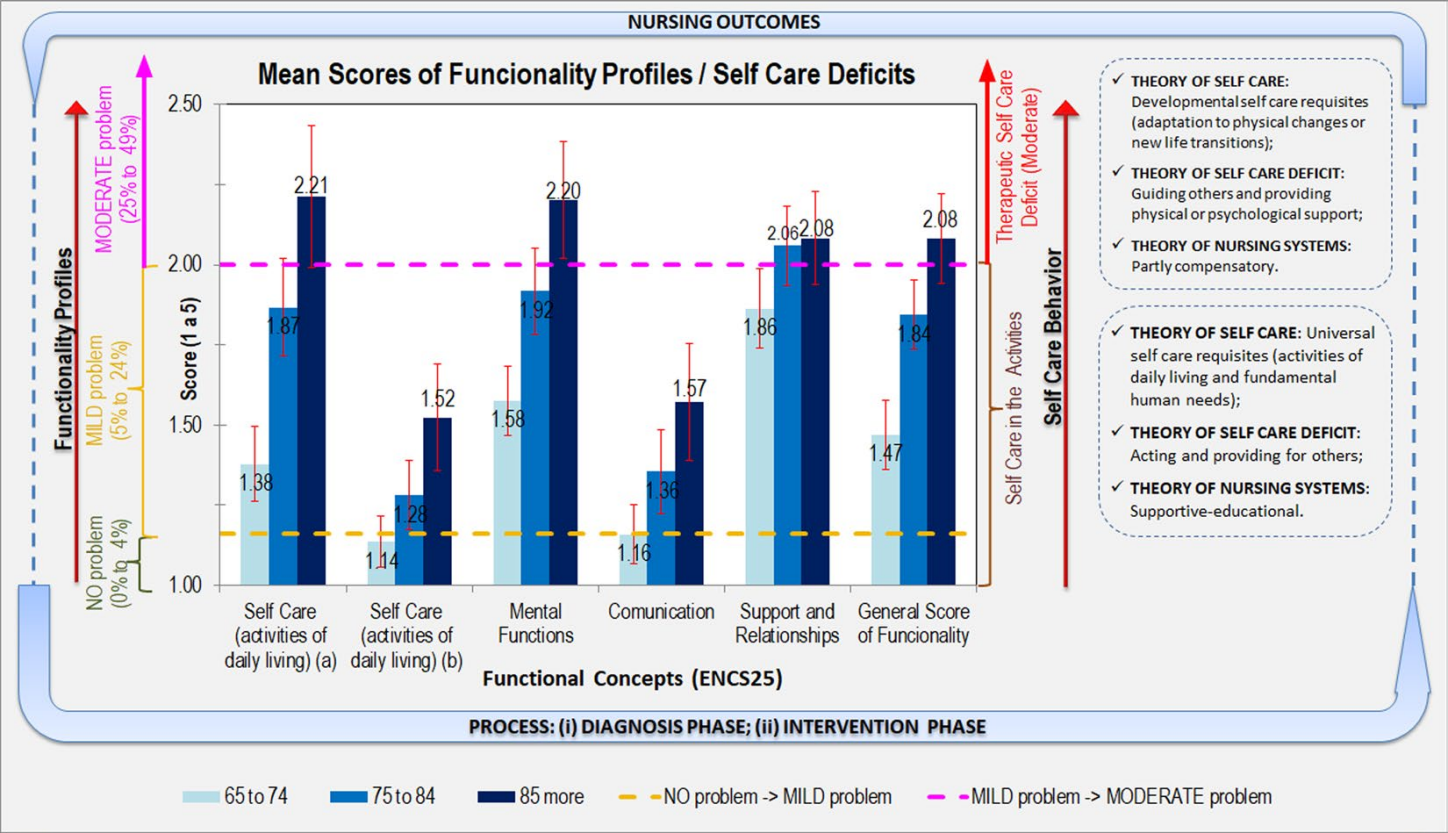
The mean scores for the functional concepts among the respondents and the correspondence in terms of nursing interventions stratified by age group, representing the elderly nursing care model based on the ENCS25.

As mentioned in the introduction section, the goal of this study was to develop a model that enables practical implementation of systematic nursing interventions based on elderly individuals’ nursing care needs identified by a study of self care behavior after evaluating the average functional profile of each of the five latent factors stratified by age group (latent factors are hereafter referred to as “functional concepts”). This model is depicted in Fig. 3 and can be used to answer the research question: “What are the predicted average nursing care needs of elderly people residing in the BAR by age group and functional concept?” The mean reference scores of the functional profiles for each functional concept are on the left lateral axis of the figure, whereas the indicator of the predicted average nursing care needs of elderly people is on the right lateral axis of the figure. The yellow and magenta dashed lines represent the reference values between the Likert scale (1 to 5) and the percentage values of 4–5% (yellow) and 24–25% (magenta) and the thresholds between the three types of functionality profiles (the group composed of the first two profiles of functionality, “no problem” and “mild problem”, and the isolated “moderate problem” profile). The red vertical lines in each bar represent the 95% CIs for the means. As an example, an 85-year-old person with a mental function score of 2.2 has a “moderate problem” functional profile, which is at the level of Therapeutic Self Care Deficit (Moderate). Thus, nursing interventions become a requirement for people when they are incapacitated or limited with regard to performing self care. These interventions are listed in the Therapeutic Self Care Deficit (Moderate) area of the Figure. In sum, the final goal of this model is to improve people’s profiles of functionality by applying necessary nursing interventions and then measuring nursing-sensitive outcomes by comparing the profiles of functionality before and after implementation of the nursing interventions. These interventions should lead to the notion of “independence”, which may be regarded as the empowerment of persons’ functional capacity on reducing their incapacities and raising their self-determination for a better management of their health processes^23^.

## Discussion

Five functional concepts (i.e., “latent factors” in Table 1) were extracted from the EFA. The first concept, “SC-ADL(a)”, was generally associated with activities of daily living related to personal care. The second concept, “SC-ADL(b)”, consisted of activities of daily living such as eating, drinking and toileting. The third concept, “Mental functions”, encompassed elements referring to orientation, attention, memory and human consciousness. The fourth concept was called “Communication” because it encompassed related elements such as dialogue (i.e., talking, conversing and communicating by sending and receiving messages). Finally, the fifth concept, “Support and relationships”, was associated with aspects related to health professionals, personal care providers/personal assistants and friends. These results suggest that the aspects related to the first concept stand out from the others because they explain the most variance compared with the four remaining concepts. With respect to activities of daily living, Sau *et al*.^24^ claim that the ability to perform ADLs can help the reduction of the likelihood of loneliness. The authors also state that ADL activities play an essential role in maintaining older adults’ mobility, thus contributing to a healthy aging. The model focuses more on ADLs rather than any other work because such activities are commonly carried out by people throughout their lifetimes, helping them to spend their free time and to find some meaning in their lives.

The *fsw* values listed in Table 1 were examined along with the mean values because high mean values associated with low *fsw* values reveal functional problems. However, this relationship had little effect on the calculation of the corresponding functional concept score. For example, the functional profile “moderate problem” for “Friends” revealed some severity with regard to relationships with friends, which is an obvious aspect for people who live in a large area with a low population density (an average distance of 20 km to 120 km between villages and a scarce and inefficient public transportation network). This area creates traveling difficulties for elderly people and encourages isolation from friends, relatives (who are the most important figures within elderly social networks) and the community. As a result, negative consequences such as loneliness emerge^25^. Another interesting aspect was to the low mean values for the items “Eating” and “Drinking” and the corresponding high *fsw* values. Accordingly, we cite Cardoso *et al*.^26^, who stated that eating ability is the last ability to be compromised in elderly individuals because dependencies emerge in reverse order compared to childhood, where eating is the first ability acquired by humans.

The functional profile of each person is interrelated with his or her sociodemographic context as well as the overarching biological, cultural and environmental characteristics of society; this aspect was generally observed in the present study, which corroborates the reports of other authors^27,28^. The current sample followed the trend observed in the scientific literature of predominantly female subjects, particularly in the older population group, reflecting a phenomenon known as the “feminization of old age”^29^. Table 3 shows that the functional profile “no problem (0–4%)” had a greater proportion of the youngest participants, which has been observed in other studies^4^. The same is true of people with the highest education levels because literacy helps people better and more effectively cope with their health/disease processes (c.f., Kimberly Parr^30^ and Abalo *et al*.^28^).

Figure 2 suggests that the most likely GPF up to 74 years of age should be “no problem” and that “mild problem” should be the most likely profile after the age of 74 years, resulting in a progressive decrease in GPFs with age, which is consistent with the findings of Lopes *et al*.^9^, Abalo *et al*.^28^ and Lesende *et al*.^5^. These results answer the research question: “What is the effect of age on the GPFs of people aged 65 and older who reside in the BAR?” Note that this question was reformulated because gender was not significant in the model.

Regarding the predictions of the nursing care needs of elderly individuals based on self care behaviors, the responses can be analyzed by examining Fig. 3. The mean functional scores obtained for the concepts “Self care (b)” and “Communication” (the left axis of Fig. 3) fall under the “Self care in activities” level of nursing care needs (the right axis of Fig. 3). For example, a person with signs of inadequate water intake or some difficulty in understanding written or spoken directives may have slight difficulty with the universal self care requisites and require nursing interventions at the supportive-educational level. Therefore, a nurse will teach and supervise activities even if the person is able to perform self care. For the concepts “Self care (a)”, “Mental functions” and “General Score of Functionality” (that allows establish GPFs), the mean scores observed for the oldest age group reached the “Therapeutic Self Care Deficit (Moderate)” level of nursing care needs (i.e., when an individual needs nursing care to help him or her with tasks that he or she is unable to accomplish independently and be compensated for such care through the nursing system). These effects can assume the forms of wholly compensatory support, partly compensatory support and educational support. The other two age groups fall under the “Self care in activities” level on average. Regarding the “Support and Relationships” functional concept (which refers to family, friends, neighbors and health professionals), the two older groups reached the “Therapeutic Self Care Deficit (Moderate)” level of nursing care needs. However, the group of 65- to 74-year-old individuals was also close to this threshold, showing that among the five functional concepts identified in this article, this concept is the most relevant for determining the nursing interventions to adopt in the self care of the studied population. Moreover, the context in which people live may be a barrier rather than a facilitator of functionality^6^, preventing the various aspects of people’s lives to converge as a whole^31^. In this situation, the therapeutic requirement of self care consists of a set of nursing interventions, such as guiding others and providing physical or psychological support, applied over time because a person may already have needs beyond his or her capacity. The model shown in Fig. 3 allows answering the research question: “What are the predicted average nursing care needs of elderly individuals residing in the BAR by age group?”.

In this study, no elderly nursing care needs at the “severe or complete self care deficit” level were identified. In summary, the ENCS25 model described in Fig. 3 (i) assesses functional capacity to identify problems (care requirements), (ii) identifies appropriate self care behavior to determine, plan and control nursing care needs and (iii) implements the nursing process based on Orem’s work across different operationalization phases^13^. On the other hand, monitoring of the effectiveness of nursing interventions to quantify health outcomes is highlighted from the perspective of improving the quality of nursing care provided, based on various “diagnosis” and “intervention” phases (see Fig. 3), and such monitoring is essential to measure the results of nursing interventions^32^.

## Conclusions

This study proposes a conceptual framework to evaluate nursing interventions according to self care deficits.

Regarding the contributions of the present study to clinical practice, this model may be integrated at the levels of data collection, care planning, intervention implementation and intervention evaluation. Thus, the model will be useful for supporting decision-making in the context of nursing care planning and resource management. The model will also allow improvement of quality standards in the provision of nursing care by systematically evaluating the results of nursing care, increasing user satisfaction and achieving health and efficiency gains based on the rule “better to know them to better care for them”. With regard to policy decisions, nursing care programs aimed at improving the self care behavior of people aged 65 and older should be developed.

In our opinion, our model contributes to the efficacy of nursing interventions, as it allows the identification of the effectiveness of nursing care along various “diagnosis” and “intervention” phases, always taking into account the persons’ real health condition, emphasizing that the “*outcome*” of such interventions always result in “*value for the elderly’s health*”^33^.

### Limitations

This study has the following limitations. The most important limitation is the low number of respondents (351, although the expected number of respondents was 470), especially due to the lower level of participation among health professionals. Since this study was carried out in a region of Portugal’s main territory with special demographic and geographic characteristics, the results may not be generalizable to other regions or to the entire country.

## Data Availability

Correspondence and requests for materials should be addressed to HO (email: hjmo@lx.it.pt).
